# Characterization of Chromosome Inheritance of the Intergeneric BC_2_ and BC_3_ Progeny between *Saccharum* spp. and *Erianthus arundinaceus*

**DOI:** 10.1371/journal.pone.0133722

**Published:** 2015-07-21

**Authors:** Yongji Huang, Jiayun Wu, Ping Wang, Yanquan Lin, Cheng Fu, Zuhu Deng, Qinnan Wang, Qiwei Li, Rukai Chen, Muqing Zhang

**Affiliations:** 1 Key Lab of Sugarcane Biology and Genetic Breeding, Ministry of Agriculture, Fujian Agriculture and Forestry University, Fuzhou, China; 2 Guangdong Key Laboratory of Sugarcane Improvement and Biorefinery, Guangzhou Sugarcane Industry Research Institute, Guangzhou, China; 3 Guangxi Collaborative Center for Sugarcane & Cane Sugar Industries, Guangxi, China; United States Department of Agriculture, UNITED STATES

## Abstract

*Erianthus arundinaceus* (*E*. *arundinaceus*) has many desirable agronomic traits for sugarcane improvement, such as high biomass, vigor, rationing ability, tolerance to drought, and water logging, as well as resistance to pests and disease. To investigate the introgression of the *E*. *arundinaceus* genome into sugarcane in the higher generations, intergeneric BC_2_ and BC_3_ progeny generated between *Saccharum* spp. and *E*. *arundinaceus* were studied using the genomic *in situ* hybridization (GISH) technique. The results showed that the BC_2_ and BC_3_ generations resulted from n + n chromosome transmission. Furthermore, chromosome translocation occurred at terminal fragments from the *E*. *arundinaceus* chromosome in some progeny of *Saccharum* spp. and *E*. *arundinaceus*. Notably, the translocated chromosomes could be stably transmitted to their progeny. This study illustrates the characterization of chromosome inheritance of the intergeneric BC_2_ and BC_3_ progeny between *Saccharum* spp. and *E*. *arundinaceus*. This work could provide more useful molecular cytogenetic information for the germplasm resources of *E*. *arundinaceus*, and may promote further understanding of the germplasm resources of *E*. *arundinaceus* for sugarcane breeders to accelerate its progress in sugarcane commercial breeding.

## Introduction

Sugarcane (*Saccharum* spp.) is a large perennial grass that is indigenous to tropical and subtropical regions. As the most important sugar-producing crop worldwide, sugarcane has significant potential to contribute to the global sugar security and produces approximately 75% of the world’s raw sugar [1]. In addition, as a C_4_ plant, sugarcane is an efficient crop in converting solar energy into chemical energy. Therefore, it has been heralded as an alternative source of fuel and petrochemical feedstock for the production of first-generation bioethanol to alleviate the current energy crisis [2].

The genus *Saccharum* is an important member of the *Poaceae* family that consists of six species, including *S*. *officinarum*, *S*. *spontaneum*, *S*. *robustum*, *S*. *barberi*, *S*. *sinense*, and *S*. *edule*. Modern sugarcane cultivars are highly complex aneupolyploids, and most are primarily derived from interspecific hybridization between *S*. *officinarum* (2n = 80) and *S*. *spontaneum* (2n = 40–128) through nobilization [3]. This term was first coined by Dutch breeders Jesweit in Java during the early 1900s to denote the process of introgression of *S*. *spontaneum* into *S*. *officinarum* following hybridization and successive backcrossing. During the nobilization process, interspecific F_1_ hybrids were obtained from crosses between *S*. *officinarum* as the female parent and *S*. *spontaneum* as the male parent, and then were repeatedly backcrossed to *S*. *officinarum* as the female parent. Using this approach, progeny conserve the entire genome of *S*. *officinarum* in the first interspecific cross (F_1_) and the first backcross (BC_1_) [4]. Hence, the chromosome inheritance of progeny in F_1_ and BC_1_ exhibits 2n + n transmission. This not only allows for a quick recovery of the high sugar content from *S*. *officinarum*, but also integrates resistance genes to biotic and abiotic stresses from *S*. *spontaneum* [5]. Jesweit succeeded in the selective breeding of some new cultivars with high resistance to disease, and significantly contributed to the perseverance through the sugar crisis in Java at that time due to disease outbreaks [2]. POJ2878, hailed as the “wonder cane”, is one of the most successful examples of the utilization of nobilization.

However, due to the frequent utilization of a limited number of progenitors in sugarcane breeding programs, modern sugarcane cultivars have given rise to a sharp decline in genetic diversity [6,7]. Genetic erosion renders sugarcane increasingly vulnerable to resistance against biotic and abiotic stresses. As a result, the genetic potential for yield and quality improvement has hardly allowed for any advancement in the past several decades. Therefore, options for remedying the growing concern of a dearth of genetic variation has become an urgent and necessary task for sugarcane breeders. One efficient approach for combating this issue is by tapping into wild relatives to introduce favorable genes for increased productivity and better adaptability to a wide large range of growing conditions as well as providing more robust disease resistance. The genus *Saccharum* together with the four related genera, namely *Erianthus*, *Miscanthus*, *Narenga*, and *Sclerostachya*, comprise the “*Saccharum* complex” [8]. These four related genera serve as a rich gene pool for sugarcane improvement with tolerance to abiotic stresses and resistance to biotic stresses. As one of the most important wild relatives of sugarcane, *Erianthus arundinaceus* (*E*. *arundinaceus*) has many superior traits for sugarcane improvement [8–12]. It has already been considered to be one of the most popular germplasm sources for crossing utilization in sugarcane improvement. However, the taxonomy of *Saccharum* and *Erianthus* had been controversial for a long time [13]. Until there is a good deal of evidence that the genetic distance is large between *Saccharum* and *Erianthus*, according to morphological characteristics, chromosome number, and phylogenetic relationship, the evidence sheds light on the taxonomic relationship between *Saccharum* and *Erianthus* [8,14–17]. Despite the large genetic distance between *Saccharum* and *Erianthus*, genuine intergeneric F_1_ hybrids and their derivatives have been successfully generated in the past [9,11,18–21].

In sugarcane, there are various types of chromosome transmission such as n + n, 2n + n, n + 2n, and 2n + 2n [19,21–26]. Compared to the common type of chromosome transmission (n + n), the other three specific types of chromosome transmission (2n + n, n + 2n, and 2n + 2n) are derived from unilateral and bilateral sexual polyploidization. In the plant kingdom, sexual polyploidization, leading to unreduced gametes (2n gametes) with the somatic chromosome number rather than the gametophytic number (n gamete), is generally believed to be the predominant mechanism of polyploidization [27,28]. Based on the different gametogenesis, the unreduced gametes are divided into 2n egg gametes and 2n male gametes. 2n egg gametes typically originate from the unreduced ovule during megasporogenesis, whereas 2n male gametes are the result of the unreduced pollen during microsporogenesis [29]. The consequence of unilateral sexual polyploidization is 2n + n (result from the fertilization of unreduced ovule by normal haploid pollen) or n + 2n (result from the fertilization of normal ovule by unreduced pollen), while the result of bilateral sexual polyploidization is 2n + 2n (result from the fertilization of unreduced ovule by unreduced pollen). Several mechanisms have been described that the types of meiotic abnormalities responsible for the production of 2n gametes. Due to the different parental heterozygosity rate that each mechanism transmits to the progeny, the genetic consequences of different types of 2n gametes formation are highly divergent [28]. Hence, the use of 2n gametes, resulting in the different types of chromosome transmission during the establishment of sexual polyploids, is of prime importance to develop and conduct breeding strategies for crop improvement [30]. Indeed, it has already been proven effective for improvement of crops such as lily, potato, banana, and citrus [31–38]. In nobilization of *S*. *spontaneum*, the utilization of 2n gametes transmission from *S*. *officinarum* in interspecific crosses with *S*. *spontaneum* is such a typical example of the cytological peculiarity of 2n gametes.

Genomic *in situ* hybridization (GISH) is a powerful molecular cytogenetic tool to unravel the chromosome composition for the detection of different chromosomes sets derived from two or more distinct species and even recombinant chromosome segments in allopolyploids. During allopolyploid speciation and evolutionary process, the occurrence of chromosomal rearrangement is common, such as translocation and inversion. So far, this molecular cytogenetic technology has been widely used in investigating the chromosome composition and chromosomal translocation in a wide range of natural allopolyploids or artificial polyploid progeny [39–46]. Thus, knowledge of inferring the chromosome transmission from the chromosome composition in allopolyploid will make it possible to implement a strategy for developing useful varieties through breeding. Using GISH, much insight has been gained into sugarcane chromosomal inheritance and genomic recombination over the past several decades [11,19–21,24]. A previous study indicated that modern sugarcane cultivars possess approximately 120 chromosomes, with 70–80% derived from *S*. *officinarum*, 10–20% from *S*. *spontaneum*, and a few chromosomes derived from interspecific recombination [24].

In this study, two generations, including nine BC_2_ progeny and eight BC_3_ progeny, were characterized by GISH. The objectives were as follows: (1) to determine the chromosome transmission in these two generations, which can provide a reference for breeding strategies for further deployment of genes and traits from *E*. *arundinaceus*; and (2) to determine the presence of various types of intergeneric chromosomal translocation and obtain information on whether they can be inherited, which can provide a basic understanding for efficient utilization in sugarcane breeding.

## Materials and Methods

### Plant materials

The plant materials used in this study consisted of 17 progeny derived from two generations (BC_2_ and BC_3_) of intergeneric hybrids between *Saccharum* spp. and *E*. *arundinaceus* (Table 1). In the F_1_ generation, F_1_ hybrids between *S*. *officinarum* and *E*. *arundinaceus* were derived from crosses between Badila (*S*. *officinarum*, 2n = 80) as the female parent and HN 92–77 (*E*. *arundinaceus*, 2n = 60) or HN 92–105 (*E*. *arundinaceus*, 2n = 60) as the male parent. In the BC_1_ generation, F_1_ hybrids between *S*. *officinarum* and *E*. *arundinaceus* were used as the female parent. CP 84–1198 (2n = 120), a commercial cultivar containing germplasms from *S*. *officinarum*, *S*. *spontaneum*, *S*. *barberi* and *S*. *robustum* without contribution from *E*. *arundinaceus*, was used as the male parent [21]. In the BC_2_ and BC_3_ generation, all the female parents and the male parents are listed in detail in Table 1. Among them, ROC10, ROC20, ROC23, YT73-204, YT91-976, YT93-159, NJ57-416, and YC95-46 are the commercial cultivars containing germplasm from *Saccharum* spp. without contribution from *E*. *arundinaceus*, while “YCE” series are the progeny of *E*. *arundinaceus*. The progeny analyzed in this study were generated at the Hainan Sugarcane Breeding Station of Guangzhou Sugarcane Industry Research Institute. All plant materials were grown in the greenhouse at Fujian Agriculture and Forestry University.

**Table 1:** The intergeneric BC_2_ and BC_3_ progeny between *Saccharum* spp. and *E*. *arundinaceus*.

Generation	Progeny	Female parent	Male parent
BC_2_	YCE03-01	NJ57-416	YCE01-116 (BC_1_)
BC_2_	YCE03-06	YCE01-116 (BC_1_)	NJ57-416
BC_2_	YCE03-16	YCE01-91 (BC_1_)	ROC23
BC_2_	YCE03-168	YCE01-123 (BC_1_)	ROC10
BC_2_	YCE03-218	YT73-204	YCE01-105 (BC_1_)
BC_2_	YCE03-249	YCE01-69 (BC_1_)	YT73-204
BC_2_	YCE03-378	ROC20	YCE01-92 (BC_1_)
BC_2_	YCE04-55	YC95-46	YCE01-102 (BC_1_)
BC_2_	YCE05-179	ROC20	YCE01-134 (BC_1_)
BC_3_	YCE05-64	YT73-204	YCE03-133 (BC_2_)
BC_3_	YCE05-150	YCE03-218 (BC_2_)	ROC10
BC_3_	YCE06-61	ROC10	YCE03-01 (BC_2_)
BC_3_	YCE06-63	ROC10	YCE03-01 (BC_2_)
BC_3_	YCE06-92	YCE04-51 (BC_2_)	YT93-159
BC_3_	YCE06-111	YCE03-168 (BC_2_)	YT93-159
BC_3_	YCE06-140	YCE03-218 (BC_2_)	ROC10
BC_3_	YCE06-166	YCE03-168 (BC_2_)	YT91-976

**Note**: “YCE” series are the progeny of *E*. *arundinaceus*, the other plant materials are the commercial cultivars containing germplasm from *Saccharum* spp. without contribution from *E*. *arundinaceus*.

### Genomic *in situ* hybridization (GISH) procedure

Chromosome preparation and the GISH experiment were carried out according to the method described by D’hont et al. [9]. Genomic DNA from Badila (*S*. *officinarum*) and YN82-114 (*S*. *spontaneum*) was labelled with Biotin, the Biotin-labeled probe was detected with Avidin D, Rhodamine 600 (XRITC) and a Biotinylated anti-avidin antibody (Vector Laboratories, Burlingame, CA), respectively. Genomic DNA from HN92-77 or HN92-105 (*E*. *arundinaceus*) was labelled with Digoxigenin, and the Digoxigenin-labeled probe was detected with sheep-anti-Digoxin-FITC (Roche, Lewes, UK) and rabbit-anti-sheep-FITC secondary antibody (Roche, Lewes, UK). Chromosomes were then counterstained with 4′, 6-diamidino-2-phenylindole (DAPI) in a Vectashield anti-fade solution (Vector Laboratories, Burlingame, CA). FISH signals were captured using an AxioScope A1 Imager fluorescent microscope (Carl Zeiss, Gottingen, Germany). In this study, results are presented as the modal number and the range of chromosomes counting four to 22 metaphases for each progeny (Table 2). The images were processed using an AxioCam MRc5 and AxioVision v.4.7 imaging software (Carl Zeiss, Gottingen, Germany).

**Table 2:** Chromosome composition of the intergeneric BC_2_ and BC_3_ progeny between *Saccharum* spp. and *E*. *arundinaceus*.

Generation	Progeny	Chromosome composition	No. of chromosomes								No. of metaphases analyzed
			Modal number	Range	Modal number	Range	Modal number	Range	Recombinants	Type of recombinants	
			Total (2n cell)		*Saccharum* spp.		*E*. *arundinaceus*				
BC_2_	YCE03-01	2n = 119 = 105S + 14E	119	115–122	105	103–106	14	12–14	0	—	10
BC_2_	YCE03-06	2n = 119 = 105S + 14E	119	118–123	105	104–107	14	13–15	0	—	16
BC_2_	YCE03-16	2n = 113 = 100S + 13E	113	109–113	100	98–101	13	12–14	0	—	15
BC_2_	YCE03-168	2n = 110 = 100S + 9E + E/S	111	108–113	100	99–103	10	8–10	1	E/S	10
BC_2_	YCE03-218	2n = 107 = 97S + 10E	107	105–108	97	97–99	10	9–12	0	—	14
BC_2_	YCE03-249	2n = 110 = 97S + 13E	110	107–113	97	96–100	13	12–15	0	—	22
BC_2_	YCE03-378	2n = 121 = 104S + 16E + S/E	121	115–121	104	101–105	16	15–17	1	S/E	8
BC_2_	YCE04-55	2n = 111 = 98S + 13E	111	109–114	98	96–100	13	11–13	0	—	18
BC_2_	YCE05-179	2n = 112 = 99S + 13E	112	108–116	99	96–101	13	13–14	0	—	12
BC_3_	YCE05-64	2n = 118 = 107S + 6E + 2(E/S)+3(S/E)	118	113–118	107	103–113	6	6	5	2(E/S)+3(S/E)	4
BC_3_	YCE05-150	2n = 116 = 108S + 8E	116	114–119	108	102–111	8	8	0	—	14
BC_3_	YCE06-61	2n = 114 = 107S + 7E	114	109–115	107	102–108	7	7	0	—	20
BC_3_	YCE06-63	2n = 105 = 98S + 7E	105	101–106	98	94–99	7	5–8	0	—	9
BC_3_	YCE06-92	2n = 118 = 109S + 7E + 2(E/S)	118	115–119	109	102–112	7	7	2	2(E/S)	10
BC_3_	YCE06-111	2n = 108 = 103S + 4E + E/S	108	104–110	103	100–106	4	4	1	E/S	15
BC_3_	YCE06-140	2n = 112 = 106S + 5E + S/E	112	108–112	106	100–109	5	5–6	1	S/E	11
BC_3_	YCE06-166	2n = 110 = 105S + 5E	110	106–111	105	101–106	5	5	0	—	4

**Note:** Since small variation of chromosome counts can occur due to the loss or the overlapping of a few chromosomes from the preparation, the modal number of chromosomes and the range of total numbers of chromosomes in 2n cell are presented for the sugarcane clones analyzed. S and E indicate *Saccharum* spp. chromosome and *E*. *arundinaceus* chromosome, respectively. S/E and E/S indicate *Saccharum* spp. centromere with *E*. *arundinaceus* chromosome segment and *E*. *arundinaceus* centromere with *Saccharum* spp. chromosome segment, respectively.

## Results and Discussion

### n + n chromosome transmission in BC_2_ and BC_3_ progeny

**Fig 1:**
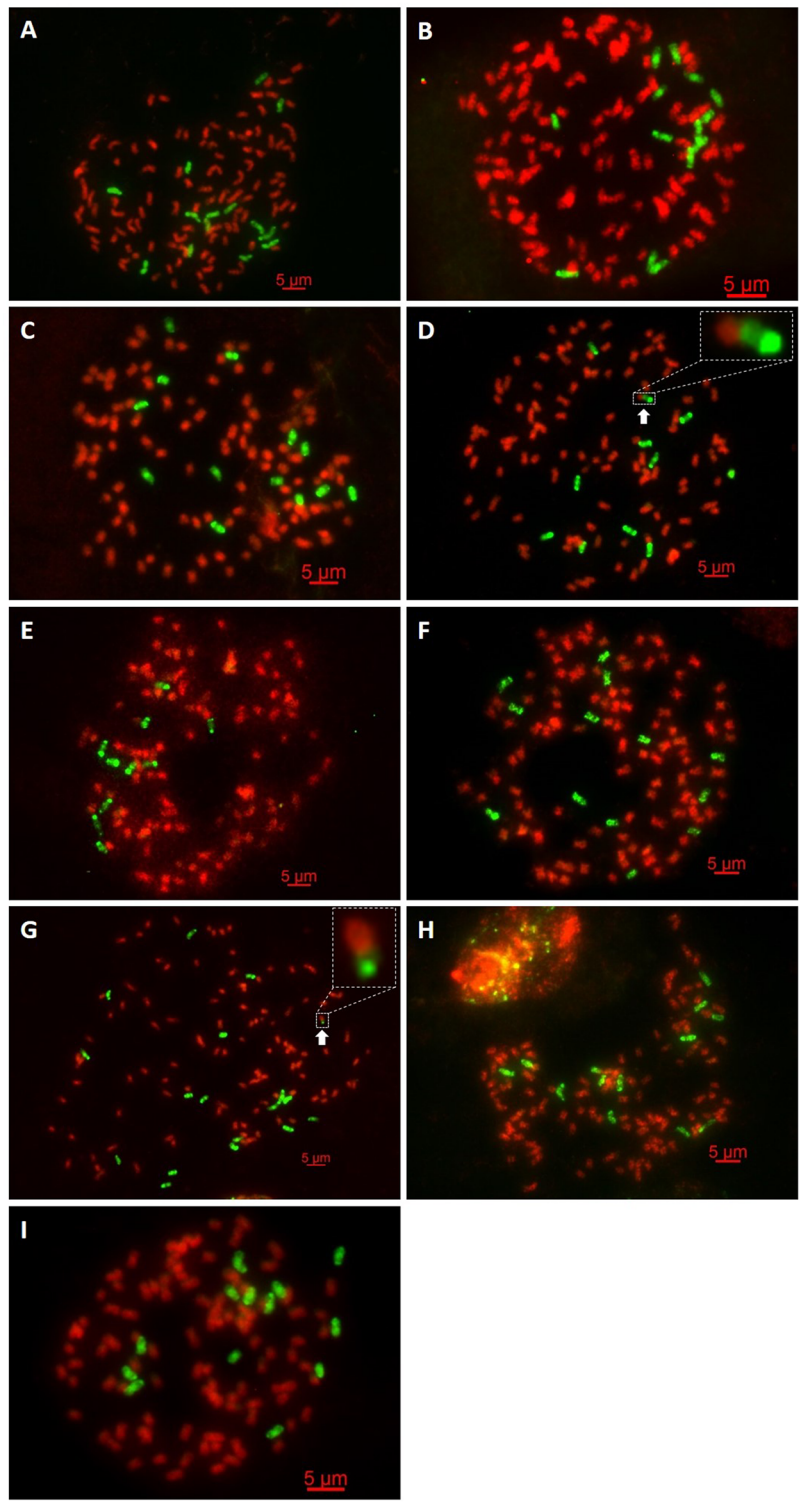
GISH analysis of the intergeneric BC_2_ progeny between *Saccharum* spp. and *E*. *arundinaceus*. *Saccharum* spp. chromosomes are visualized in red and *E*. *arundinaceus* chromosomes in green. (A) YCE03-01: 2n = 119 = 105S + 14E; (B) YCE03-06: 2n = 119 = 105S + 14E; (C) YCE03-16: 2n = 113 = 100S + 13E; (D) YCE03-168: 2n = 111 = 100S + 10E + E/S; (E) YCE03-218: 2n = 107 = 97S + 10E; (F) YCE03-249: 2n = 110 = 97S + 13E; (G) YCE03-378: 2n = 121 = 104S + 16E + S/E; (H) YCE04-55: 2n = 111 = 98S + 13E; (I) YCE05-179: 2n = 112 = 99S + 13E. The arrowheads in Fig 1D and Fig 1G show the translocated chromosome. S and E indicate *Saccharum* spp. chromosome and *E*. *arundinaceus* chromosome, respectively. S/E and E/S indicate *Saccharum* spp. centromere with *E*. *arundinaceus* chromosome segment and *E*. *arundinaceus* centromere with *Saccharum* spp. chromosome segment, respectively. Scale bars: 5 μm.

**Fig 2:**
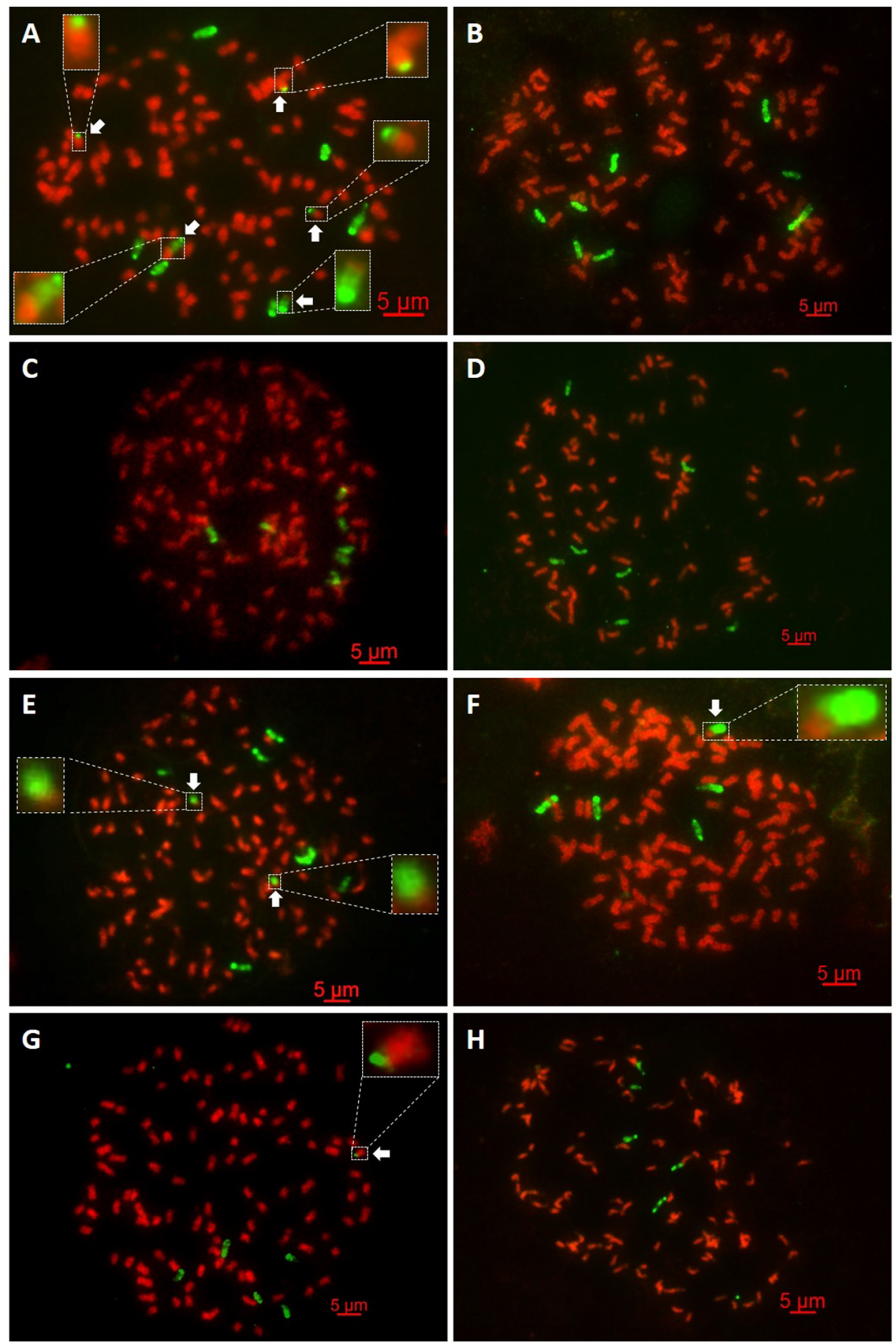
GISH analysis of the intergeneric BC_3_ progeny between *Saccharum* spp. and *E*. *arundinaceus*. *Saccharum* spp. chromosomes are visualized in red and *E*. *arundinaceus* chromosomes in green. (A) YCE05-64: 2n = 118 = 107S + 6E + 2(E/S)+3(S/E); (B) YCE05-150: 2n = 116 = 108S + 8E; (C) YCE06-61: 2n = 114 = 107S + 7E; (D) YCE06-63: 2n = 105 = 98S + 7E; (E) YCE06-92: 2n = 118 = 109S + 7E + 2(E/S); (F) YCE06-111: 2n = 108 = 103S + 4E + E/S; (G) YCE06-140: 2n = 112 = 106S + 5E + S/E; (H) YCE06-166: 2n = 110 = 105S + 5E. The arrowheads in Fig 2A, Fig 2E, Fig 2F and Fig 2G show the translocated chromosome. S and E indicate *Saccharum* spp. chromosome and *E*. *arundinaceus* chromosome, respectively. S/E and E/S indicate *Saccharum* spp. centromere with *E*. *arundinaceus* chromosome segment and *E*. *arundinaceus* centromere with *Saccharum* spp. chromosome segment, respectively. Scale bars: 5 μm.

GISH experiments of nine BC_2_ progeny revealed plants with a total chromosome complement ranging from 107 to 121 chromosomes, of which 97–105 were derived from *Saccharum* spp. and 9–16 from *E*. *arundinaceus*, respectively (Table 2, Fig 1A–1I; Figs A-I in S1 File). Since 22–35 chromosomes were derived from *E*. *arundinaceus* in BC_1_ as parents (YCE01-69, YCE01-102, YCE01-92, YCE01-134, YCE01-105 and YCE01-116) [21], these results indicate that the number of *E*. *arundinaceus* chromosomes in the BC_2_ progeny was reduced by approximately half of the *E*. *arundinaceus* chromosomes of the BC_1_ parents. For instance, YCE01-116 (BC_1_) was the male parent of YCE03-01; YCE01-116 and YCE03-01 bore 28 and 14 chromosomes derived from *E*. *arundinaceus*, respectively [21]. This indicates that the BC_2_ progeny were products of n + n transmission. GISH experiments of eight BC_3_ progeny revealed plants with a total chromosome complement ranging from 105 to 118 chromosomes, of which 98–109 were derived from *Saccharum* spp. and 4–8 from *E*. *arundinaceus*, respectively (Table 2 and Fig 2A–2H; Figs A-H in S2 File). Since 9–14 chromosomes were derived from *E*. *arundinaceus* in BC_2_ as parents (YCE03-168, YCE03-218 and YCE03-01), these results indicate that approximately half of the *E*. *arundinaceus* chromosomes of the BC_2_ parents was transmitted to the BC_3_ progeny. For example, YCE03-01 (BC_2_) was the male parent of YCE06-63; YCE03-01 and YCE06-63 bore 14 and 7 chromosomes derived from *E*. *arundinaceus*, respectively. Our results indicate that the eight BC_3_ progeny were products of n + n transmission. Piperidis et al. [19,20] reported that the similar transmission was in some different intergeneric BC_2_ and BC_3_ progeny between *Saccharum* spp. and *E*. *arundinaceus*.

In this study, YCE05-150 is a sibling line of YCE06-140 with five *E*. *arundinaceus* chromosomes. However, the number of *E*. *arundinaceus* chromosomes in YCE05-150 is eight. That is, more than half of the *E*. *arundinaceus* chromosomes in YCE03-218 was transmitted to YCE05-150. Given the results of chromosome count, the number of total chromosomes in YCE05-150, YCE03-218 (as the female parent) and ROC10 (as the male parent) were 116, 107 and 112, respectively. We can exclude the possibility that YCE05-150 was the product of 2n + n or n + 2n transmission. In fact, YCE05-150 was also the product of n + n transmission.

In nobilization of *S*. *spontaneum*, the interspecific F_1_ hybrids and BC_1_ progeny result from 2n + n transmission. The speedy process of nobilization is conducted to recover high biomass yield and sugar content from *S*. *officinarum* while retaining the stress tolerance characteristics from *S*. *spontaneum*. As a general rule, the improved varieties are obtained in the BC_2_ or BC_3_. However, the increasing times of nobilization may negatively impact the recovery of vigor and resistance to biotic or abiotic stresses. Molecular cytogenetic studies of *E*. *arundinaceus* indicated that chromosome transmission was n + n in F_1_, BC_2_, and BC_3_ generations, but was 2n + n in the BC_1_ generation [19–21]. Compared to the progress of nobilization for utilization of *S*. *spontaneum*, this slows down the progress of nobilization in utilization of *E*. *arundinaceus* and may require the improved varieties from the BC_3_, BC_4_, or even higher generations. Interestingly, Wu et al. [21] recently reported that an unexpected inheritance pattern of *E*. *arundinaceus* chromosomes resulted from more than a 2n + n transmission in the BC_1_ generation. This may lead to the presence of a larger number of new, multilocus allelic combinations and potentially creates a massive opportunity for selection of desirable traits in newly synthesized germplasm.

Smut caused by *Sporisorium scitamineum* is a destructive and worldwide disease of sugarcane, resulting in severe yield reductions and considerable loss in sugar content. Wild relatives represent potentially important sources of desirable genes for sugarcane improvement. As one of the most important wild relatives of sugarcane, *E*. *arundinaceus* has the potential to improve disease resistance in sugarcane. Introgressing from wild relatives into sugarcane is an effective approach to broadening the genetic basis of sugarcane germplasm. More strikingly, recent reports have proven that YCE05-179, a BC_2_ progeny, is resistant to sugarcane smut [47,48]. The rest of the other yet-to-be-identified progeny between *Saccharum* spp. and *E*. *arundinaceus* might provide superior lines resistant to abiotic and biotic stresses. It is necessary to screen the progeny between *Saccharum* spp. and *E*. *arundinaceus* for those most likely to improve commercial and agricultural traits.

### Chromosomal translocation in the intergeneric BC_2_ and BC_3_ progeny between *Saccharum* spp. and *E*. *arundinaceus*

The current results obtained in this study together with those present in a previous study indicate that eight progeny harbored an intergeneric chromosomal translocation between *Saccharum* spp. and *E*. *arundinaceus* (Table 2, Fig 1D, 1G; Figs D, G in S1 File; and Fig 2A, 2E, 2F, 2G; Figs A, E, F, G in S2 File). In our previous study, out of 13 BC_1_ progeny analyzed, two BC_1_ progeny (YCE01-36 and YCE01-92) both carried an intergeneric translocated chromosome, and the chromosomal translocation occurred at a terminal fragment from the *E*. *arundinaceus* chromosome [21]. In this present study, out of nine BC_2_ progeny analyzed, two BC_2_ progeny (YCE03-168 and YCE03-378) both contained one translocated chromosome, and the chromosomal translocation occurred at a terminal fragment from the *E*. *arundinaceus* chromosome (Table 2, Fig 1D and 1G; Figs D and G in S1 File). Moreover, out of eight BC_3_ progeny analyzed, there were four BC_3_ progeny (YCE05-64, YCE06-92, YCE06-111, and YCE06-140) with chromosome translocations. Piperidis et al. [20] also reported the presence of intergeneric chromosomal translocations between *Saccharum* spp. and *E*. *arundinaceus* in BC_3_. In our study, YCE06-111 and YCE06-140 possessed an intergeneric translocated chromosome, and YCE06-92 and YCE05-64 carried two and five translocated chromosomes, respectively. The chromosomal translocation occurred at the terminal fragment from *E*. *arundinaceus* chromosomes in all these cases (Table 2, Fig 2A, 2E, 2F and 2G; Figs A, E, F, G in S2 File). Notably, multiple chromosome translocations tend to occur in YCE06-92 and YCE05-64, indicating that these two progeny were more prone to translocation. These results revealed that chromosome breakpoint tended to occur at the terminal fragment from *Saccharum* spp. chromosome and/or *E*. *arundinaceus* chromosome. But in essence, there are basically two distinct types of recombinant chromosome in these chromosomal translocation events: (1) the recombinant chromosome of *Saccharum* spp. with terminally located fragment of *E*. *arundinaceus* (S/E); and (2) the recombinant chromosome of *E*. *arundinaceus* with terminally located fragment of *Saccharum* spp. (E/S). In both types, S/E and E/S indicate *Saccharum* spp. centromere with *E*. *arundinaceus* chromosome segment and *E*. *arundinaceus* centromere with *Saccharum* spp. chromosome segment, respectively. In the light of the GISH results in our previous and present study, the type of recombinant chromosome in YCE01-36, YCE01-92, YCE03-378 and YCE06-140 belongs to the former type, whereas that in YCE03-168, YCE06-92 and YCE06-111 belongs to the latter one. In addition, the type of recombinant chromosome in YCE05-64 is the admixture of S/E and E/S, including 2(E/S) and 3(S/E).

### Translocated chromosomes are stably transmitted to the progeny

Based on the pedigree, YCE01-92 (BC_1_) is the female parent for YCE03-378 (BC_2_), and YCE03-168 (BC_2_) is the male parent for YCE06-111 (BC_3_), respectively. In YCE01-92 there is one recombinant chromosome which is transmitted to YCE03-378. Similarly, in YCE03-168 there is also one recombinant chromosome which is transmitted to YCE06-111. That is, according to the transgenerational inheritance of the translocated chromosome, we can conclude that these translocated chromosomes could be stably transmitted to the progeny in subsequent generations. We recently reported that chromosome translocations only occur in the terminal regions and not the centromeric regions. This finding demonstrated that the terminal regions of the *E*. *arundinaceus* and/or *Saccharum* spp. chromosomes are more actively involved in translocations than the centromeric regions. It is possible that this is because intercalary translocations require more chromosome breakage events than terminal translocations, and therefore rarely occur. In addition, recombination events occur in different generations and in different progeny, suggesting that translocation events are not restricted to an individual progeny.

### Significance of intergeneric chromosome translocation for sugarcane improvement

Previous molecular cytogenetic studies have suggested that a few chromosomes were derived from interspecific recombination between *S*. *officinarum* and *S*. *spontaneum* in modern sugarcane cultivars [24]. In our study, despite a large genetic distance between *Saccharum* spp. and *E*. *arundinaceus* [15,16,49], the occurrence of intergeneric chromosomal translocations occurred within the BC_1_, BC_2_, and BC_3_ generations. These results suggest that intergeneric chromosome translocations can occur during an early generation. A considerable number of the recombinant chromosomes confirmed that homologous recombination occurs in the resulting progeny from *Saccharum* spp. and *E*. *arundinaceus*. Undoubtedly, the intergeneric chromosome translocations between *Saccharum* spp. chromosomes and *E*. *arundinaceus* chromosomes in this study represent a new genetic variation between these two genomes. From the point-of-view of sugarcane, intergeneric chromosome translocations can import *E*. *arundinaceus* chromosome segments or useful genes of *E*. *arundinaceus* into sugarcane. A translocation event can lead to a break in the genetic linkage, which increases the opportunity to segregate genetic variation and opens up the possibility for generating genetic and phenotypic novelty. Importantly, this kind of genetic variation may have a positive impact on sugarcane improvement.

## Supporting Information

S1 File GISH analysis of the intergeneric BC_2_ progeny between *Saccharum* spp. and *E*. *arundinaceus*. (A) *Saccharum* spp. chromosomes are visualized in red; (B) *E*. *arundinaceus* chromosomes are visualized in green; (C) All chromosomes are counterstained in blue; (D) A merged image is generated from the red and green channels. S and E indicate *Saccharum* spp. chromosome and *E*. *arundinaceus* chromosome, respectively. S/E and E/S indicate *Saccharum* spp. centromere with *E*. *arundinaceus* chromosome segment and *E*. *arundinaceus* centromere with *Saccharum* spp. chromosome segment, respectively. Scale bars: 5 μm.
(DOC)

S2 File GISH analysis of the intergeneric BC_3_ progeny between *Saccharum* spp. and *E*. *arundinaceus*. (A) *Saccharum* spp. chromosomes are visualized in red; (B) *E*. *arundinaceus* chromosomes are visualized in green; (C) All chromosomes are counterstained in blue; (D) A merged image is generated from the red and green channels. S and E indicate *Saccharum* spp. chromosome and *E*. *arundinaceus* chromosome, respectively. S/E and E/S indicate *Saccharum* spp. centromere with *E*. *arundinaceus* chromosome segment and *E*. *arundinaceus* centromere with *Saccharum* spp. chromosome segment, respectively. Scale bars: 5 μm.
(DOC)
