# Supplementary material for: Characterization of Chromosome Inheritance of the Intergeneric BC2 and BC3 Progeny between Saccharum spp. and Erianthus arundinaceus
Source: PLoS One. 2015 Jul 21;10(7):e0133722. doi: 10.1371/journal.pone.0133722 (PMC4510360; doi:10.1371/journal.pone.0133722)

Fig. A. YCE03-01: 2n = 119 = 105S + 14E


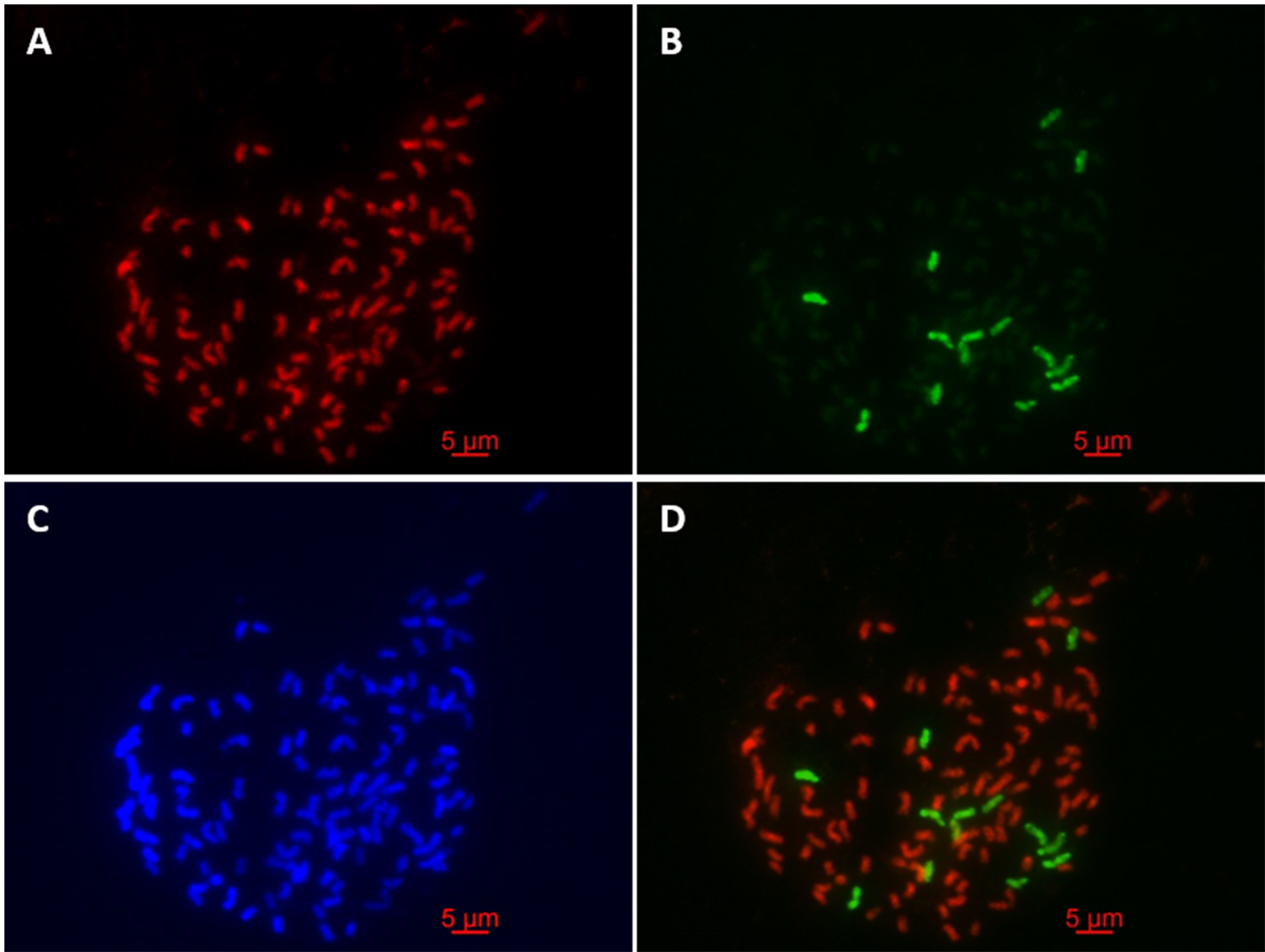


Fig. B. YCE03-06: 2n = 119 = 105S + 14E


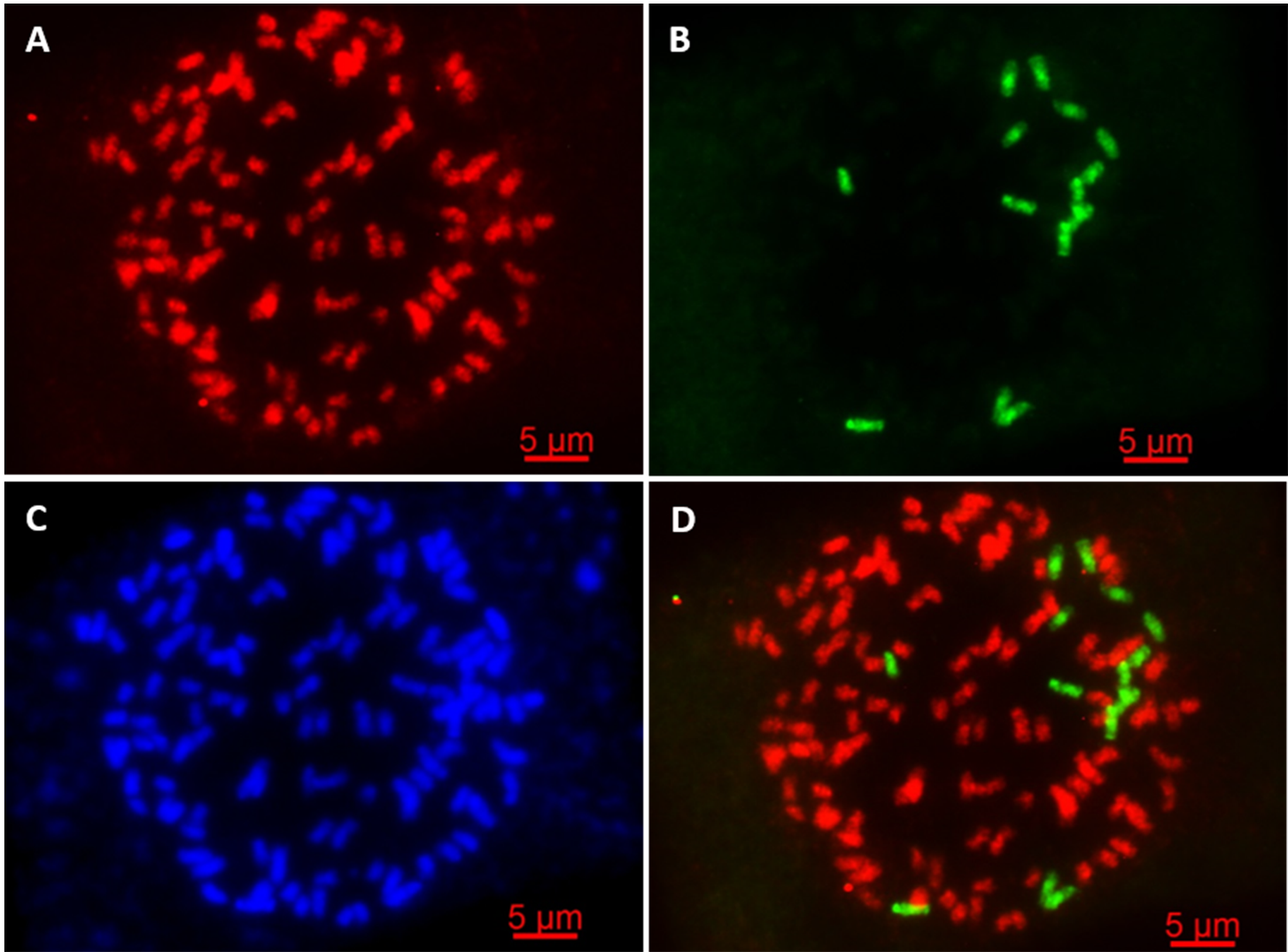


Fig. C. YCE03-16: 2n = 113 = 100S + 13E


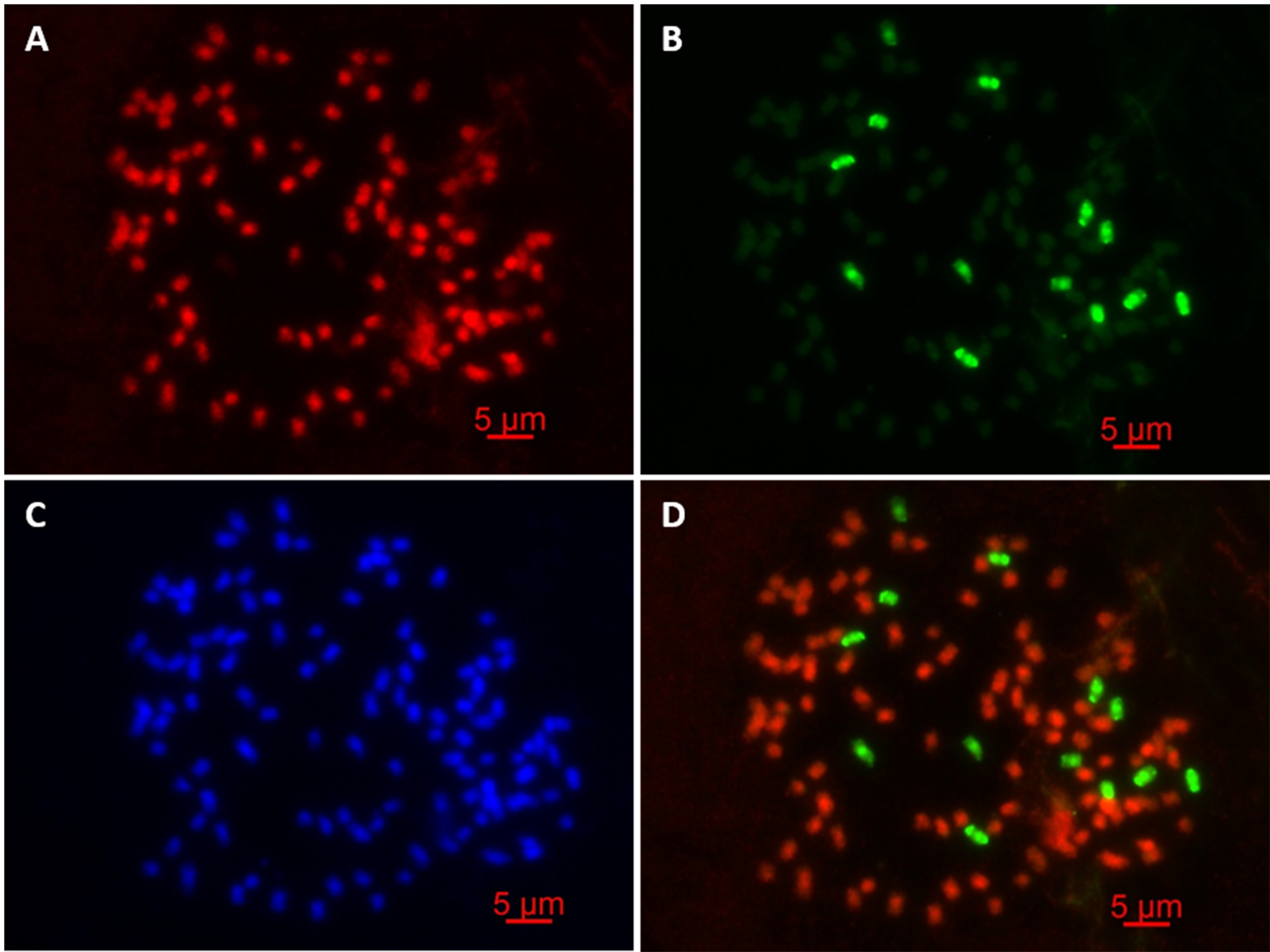


Fig. D. YCE03-168: 2n = 111 = 100S + 10E + E/S


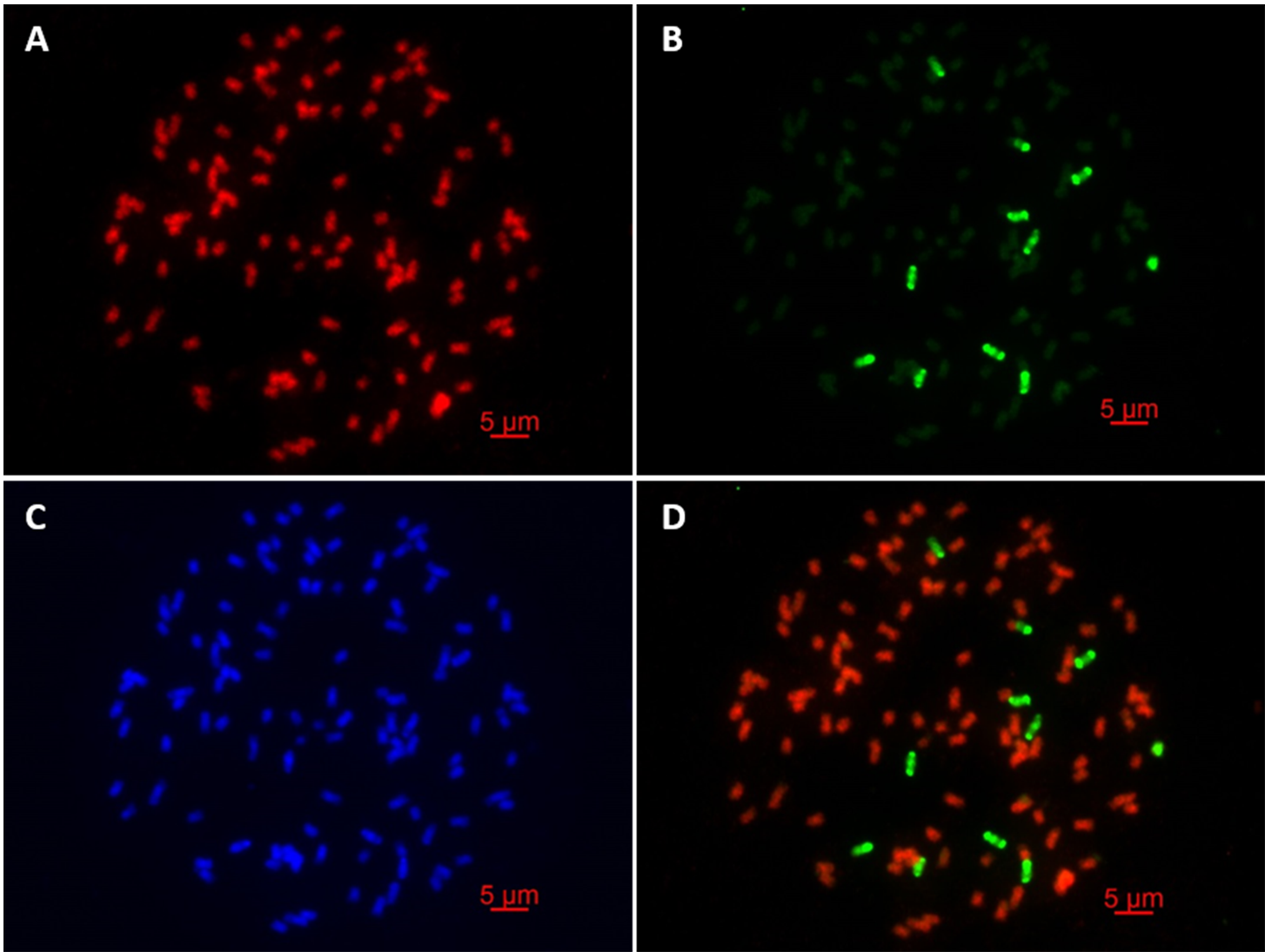


Fig. E. YCE03-218: 2n = 107 = 97S + 10E


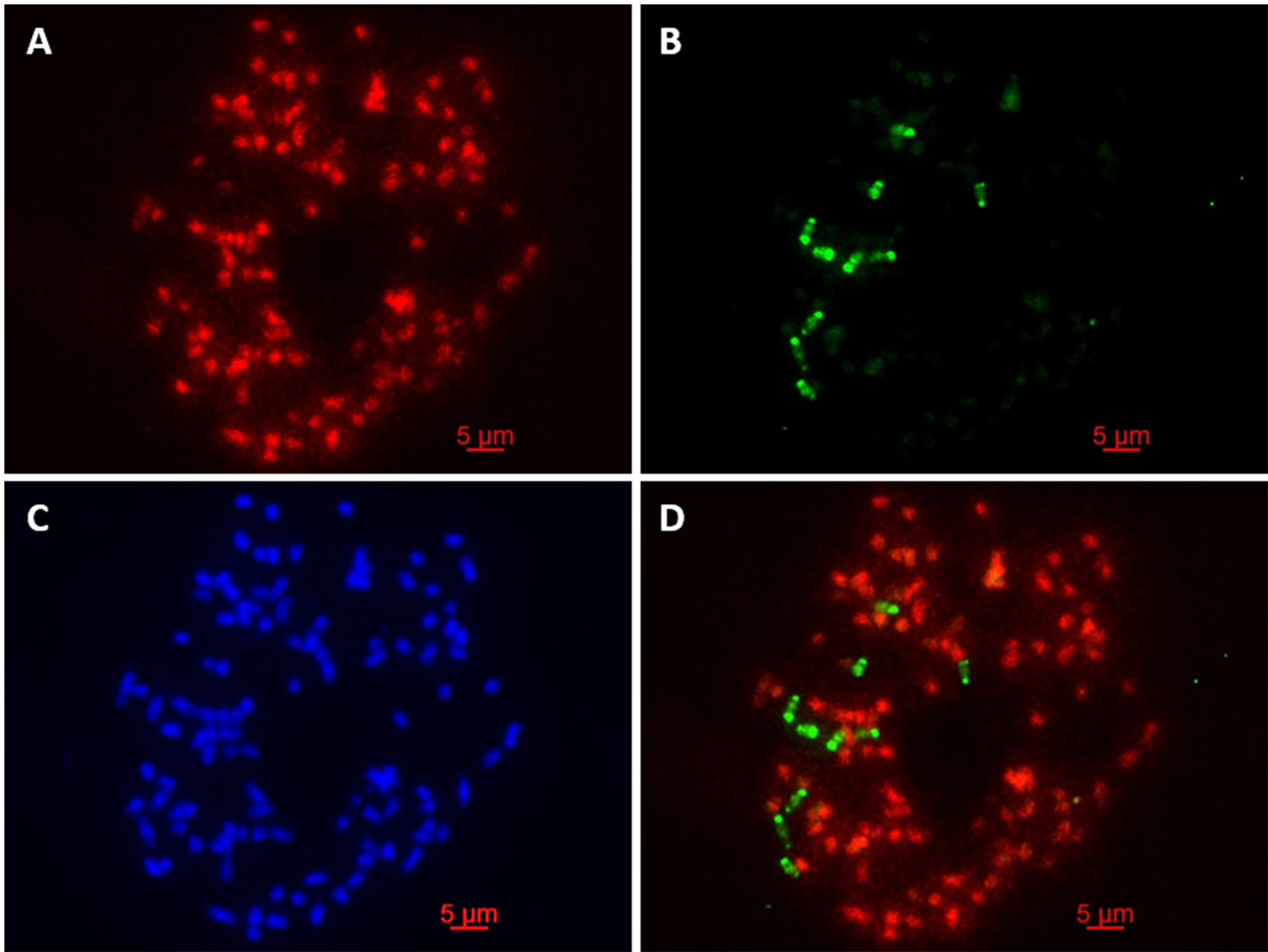


Fig. F. YCE03-249: 2n = 110 = 97S + 13E


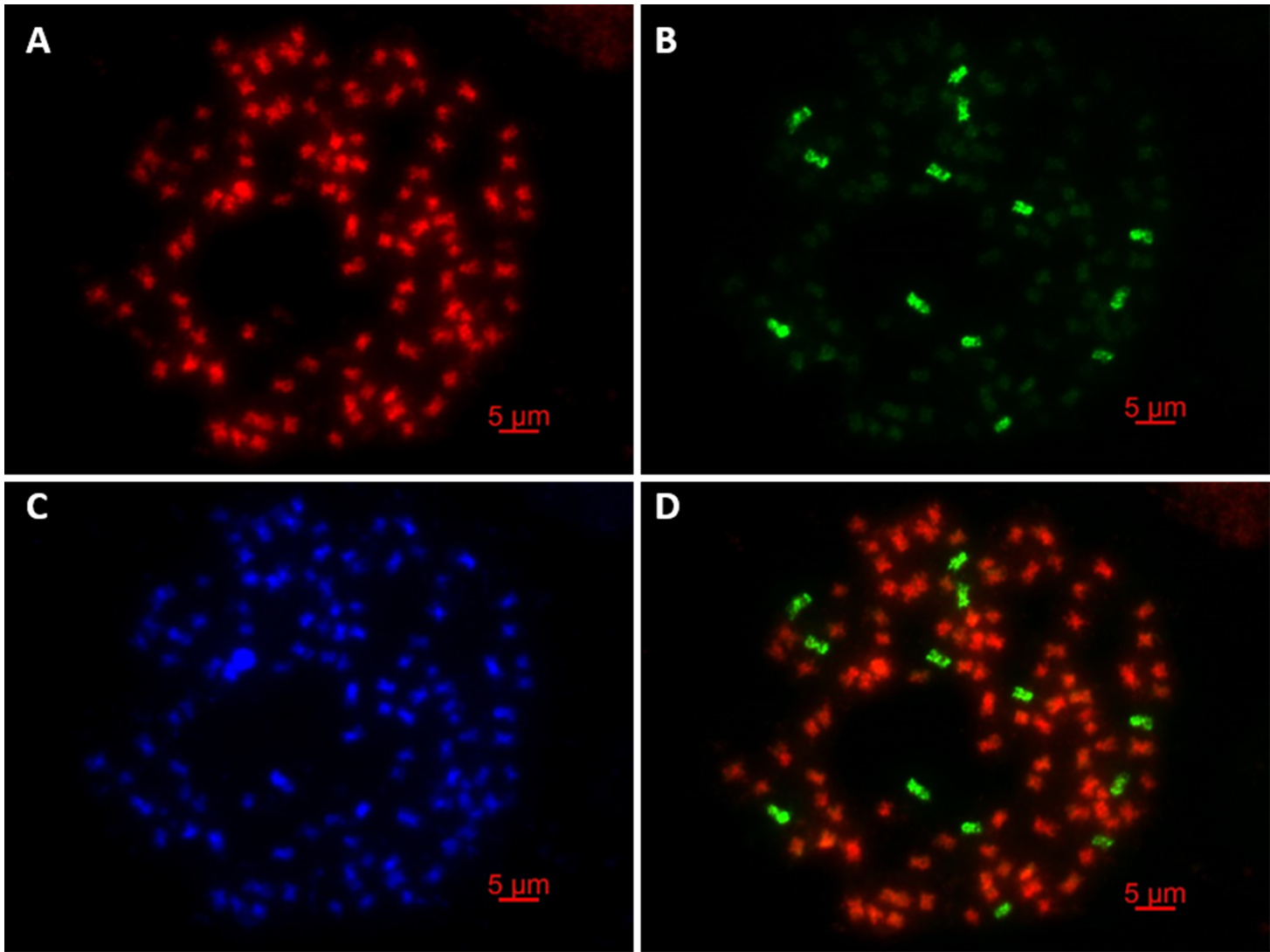


Fig. G. YCE03-378: 2n = 121 = 104S + 16E + S/E


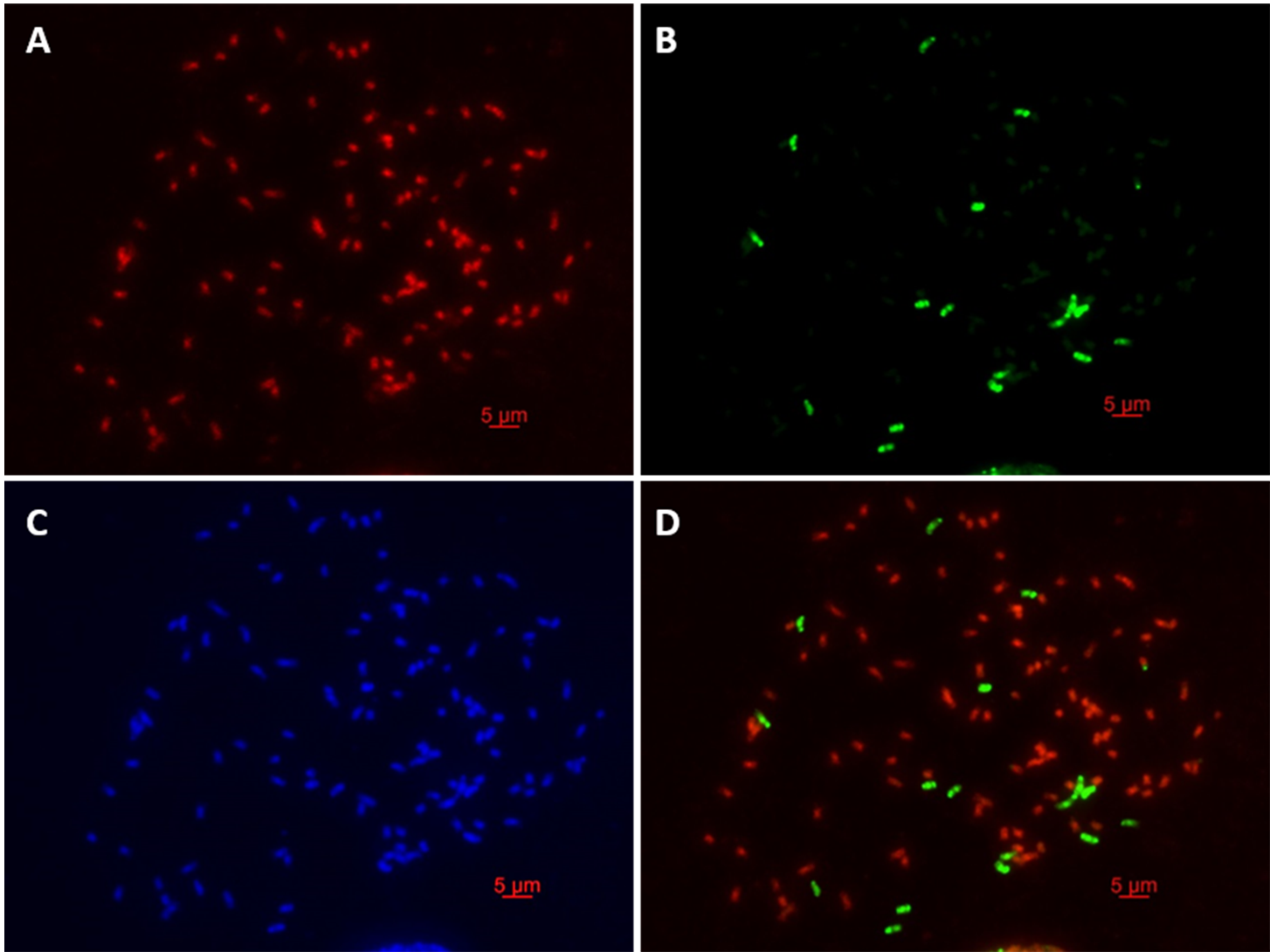


Fig. H. YCE04-55: 2n = 111 = 98S + 13E


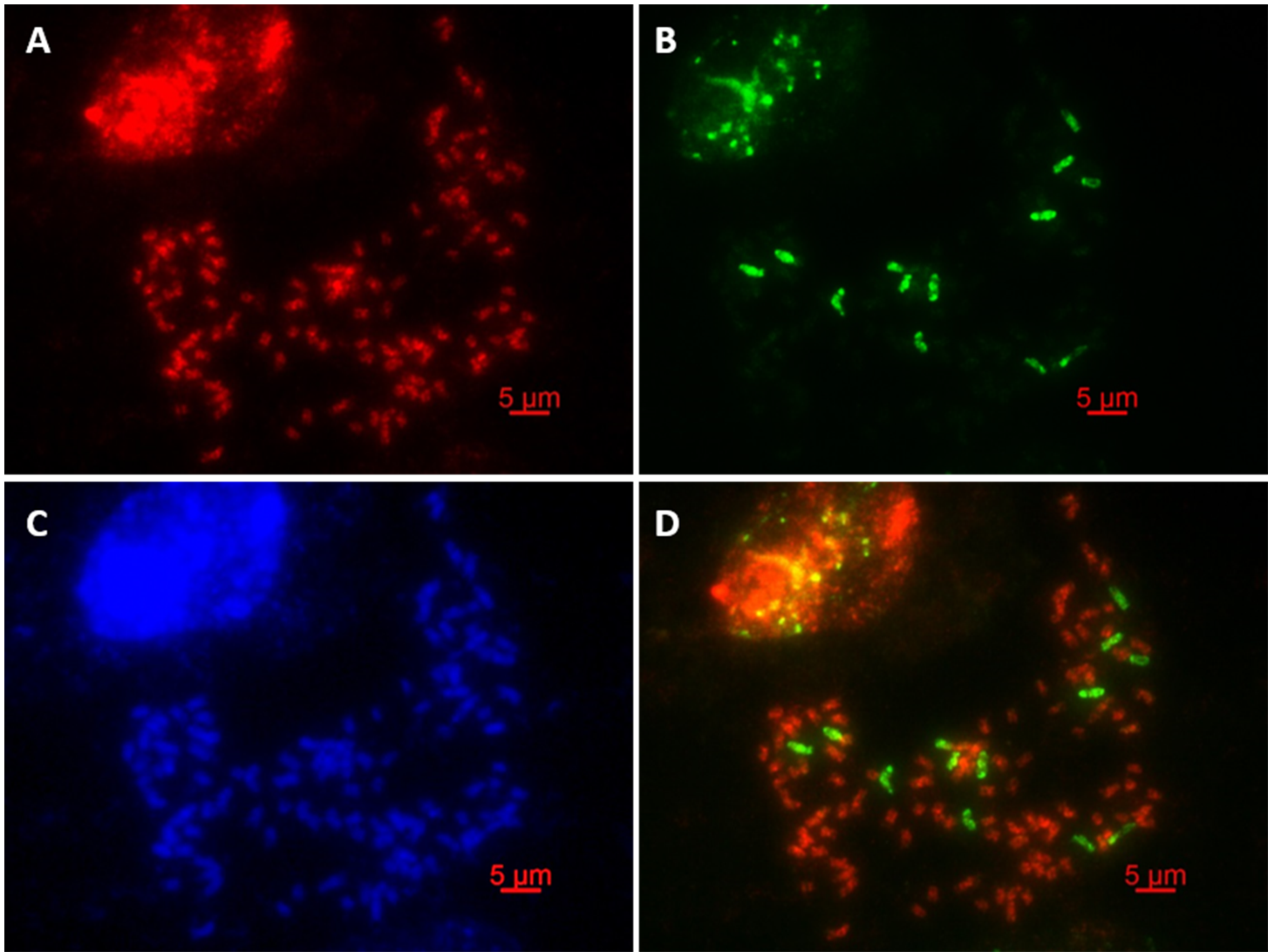


Fig. I. YCE05-179: 2n = 112 = 99S + 13E


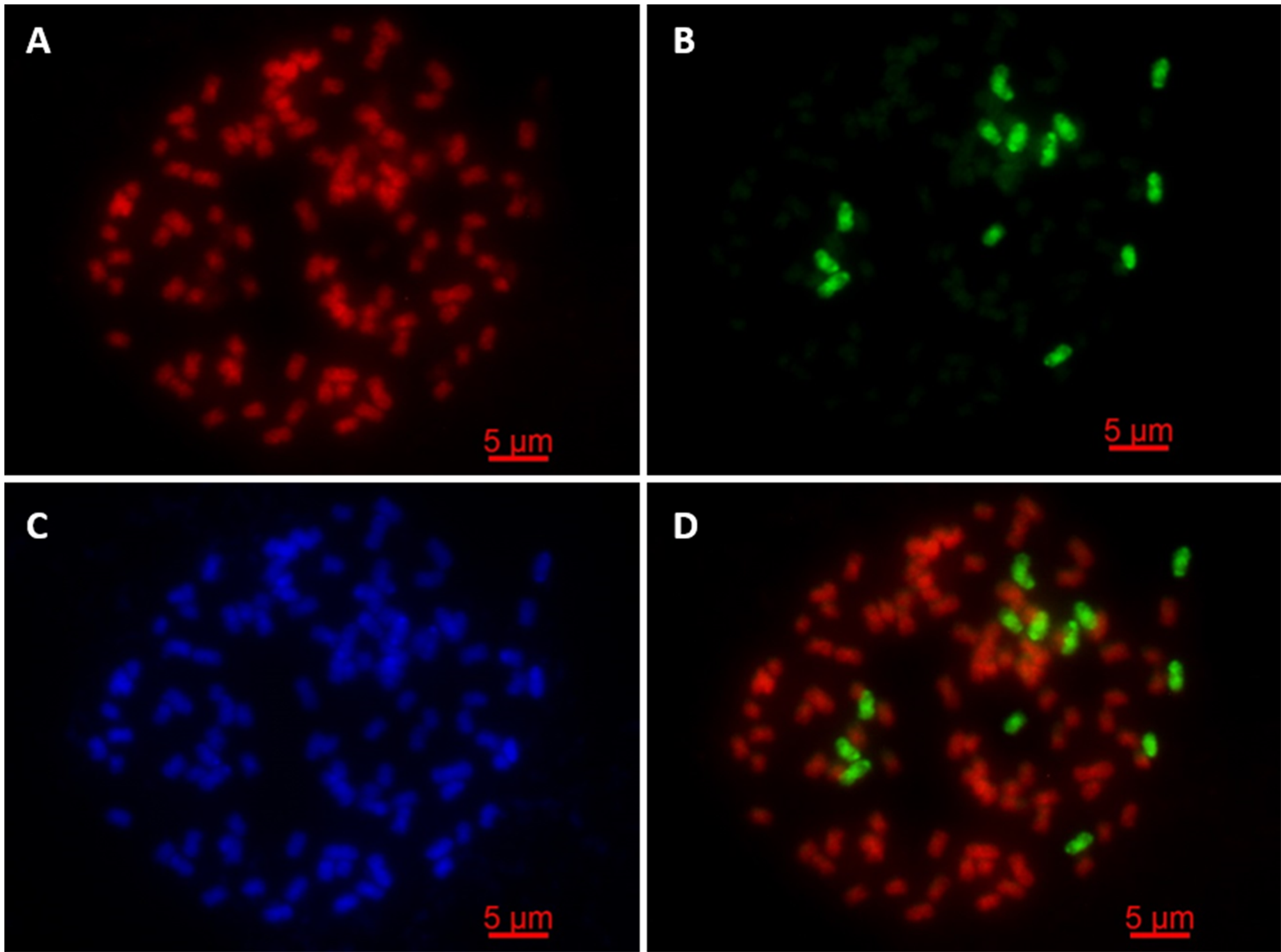

Supplement: S1 File — (A) Saccharum spp. chromosomes are visualized in red; (B) E. arundinaceus chromosomes are visualized in green; (C) All chromosomes are counterstained in blue; (D) A merged image is generated from the red and green channels. S and E indicate Saccharum spp. chromosome and E. arundinaceus chromosome, respectively. S/E and E/S indicate Saccharum spp. centromere with E. arundinaceus chromosome segment and E. arundinaceus centromere with Saccharum spp. chromosome segment, respectively. Scale bars: 5 μm. (DOC) [file pone.0133722.s001.doc]
