# Supplementary material for: Characterization of Chromosome Inheritance of the Intergeneric BC2 and BC3 Progeny between Saccharum spp. and Erianthus arundinaceus
Source: PLoS One. 2015 Jul 21;10(7):e0133722. doi: 10.1371/journal.pone.0133722 (PMC4510360; doi:10.1371/journal.pone.0133722)

Fig. A. YCE05-64: 2n = 118 = 107S + 6E + 2(E/S) + 3(S/E)


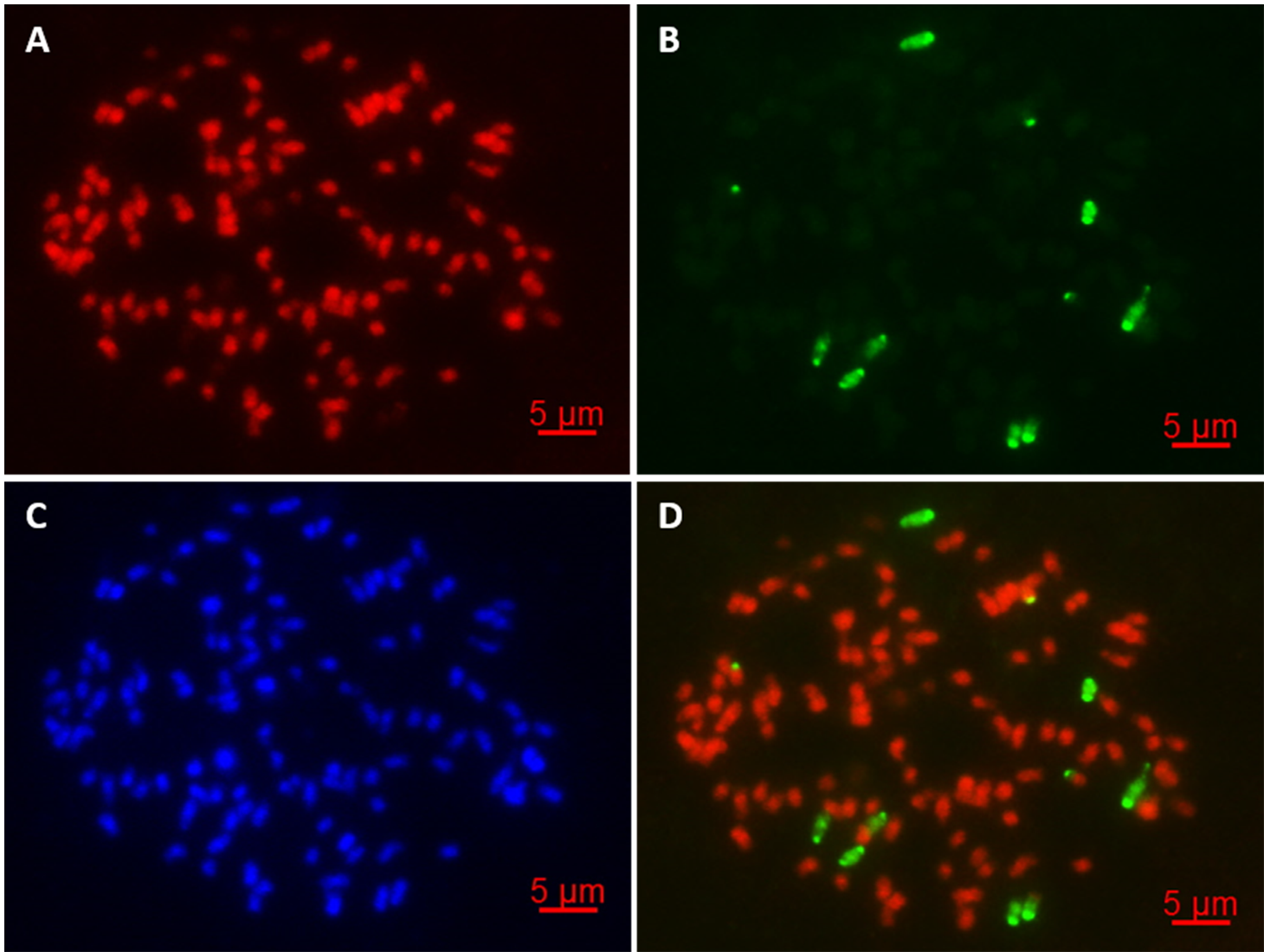


Fig. B. YCE05-150: 2n = 116 = 108S + 8E


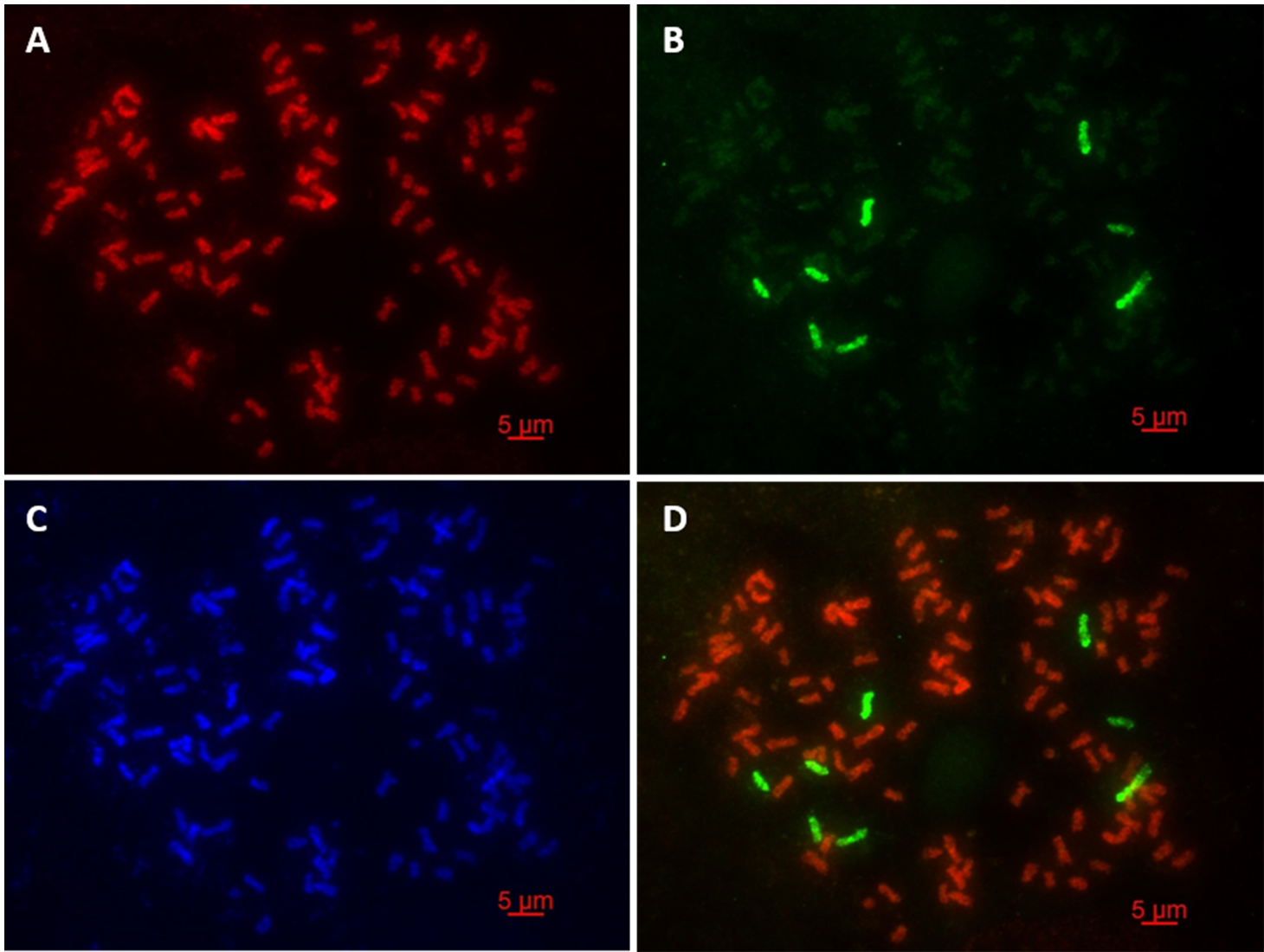


Fig. C. YCE06-61: 2n = 114 = 107S + 7E


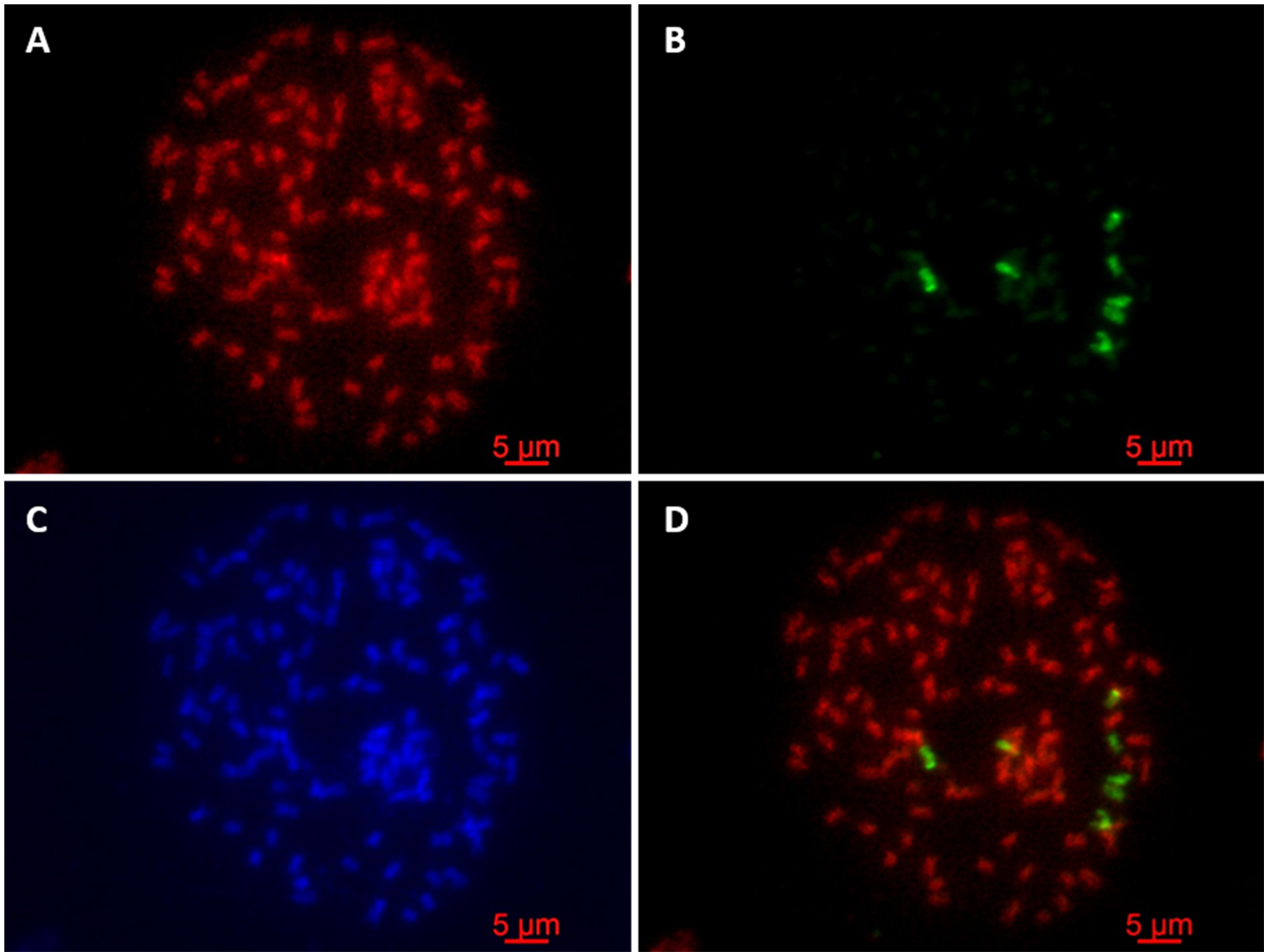


Fig. D. YCE06-63: 2n = 105 = 98S + 7E


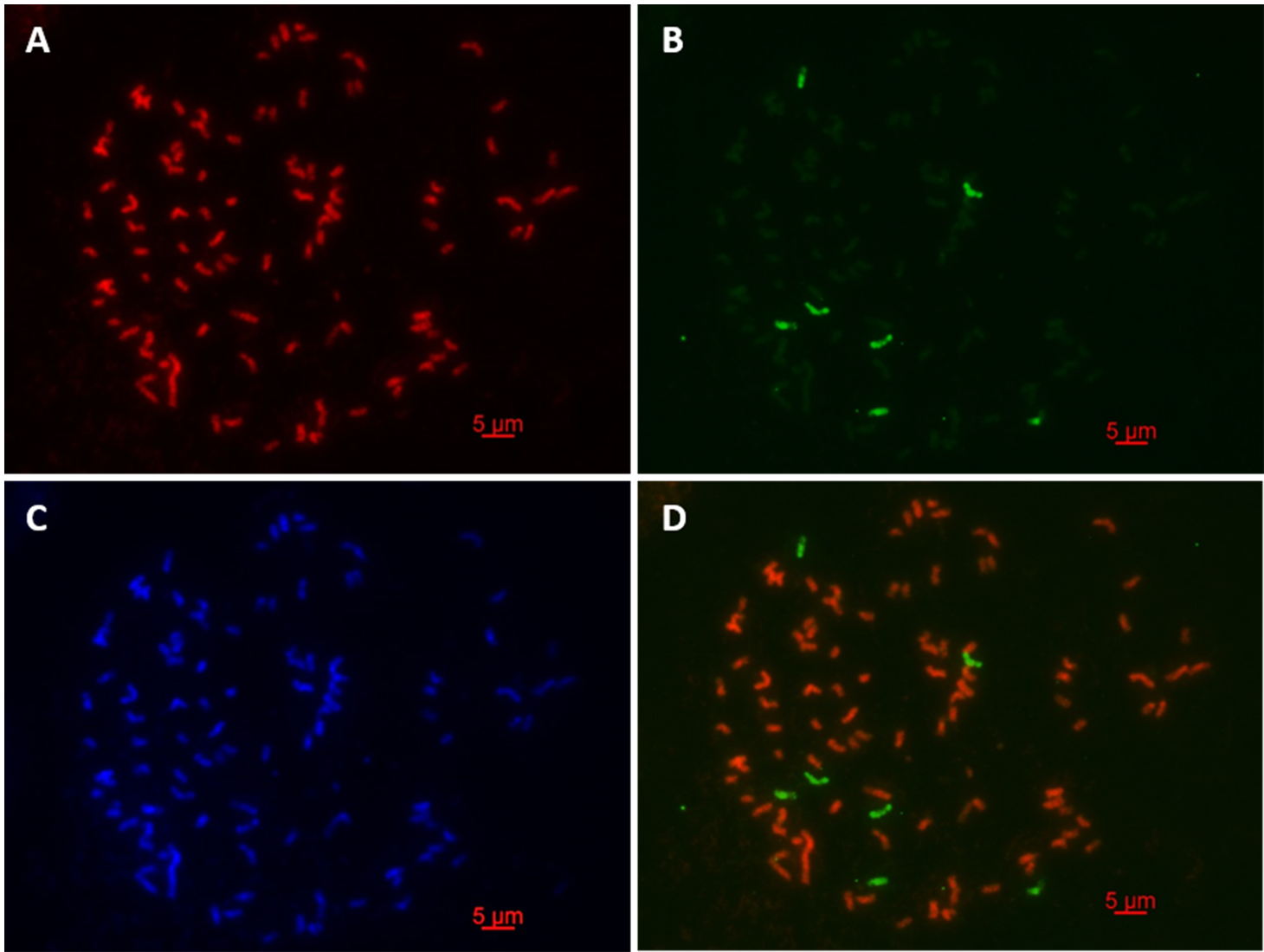


Fig. E. YCE06-92: 2n = 118 = 109S + 7E + 2(E/S)


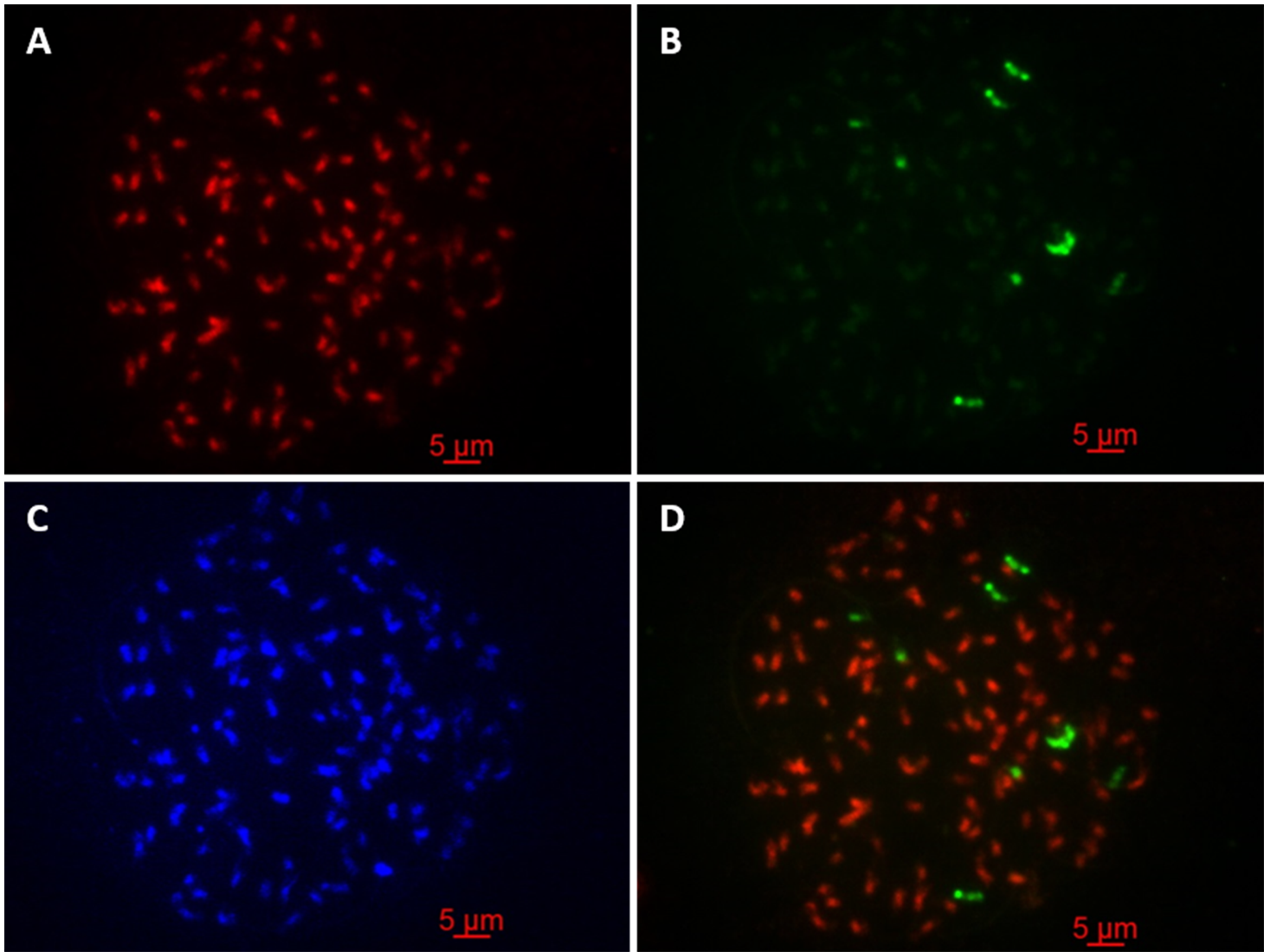


Fig. F. YCE06-111: 2n = 108 = 103S + 4E + E/S


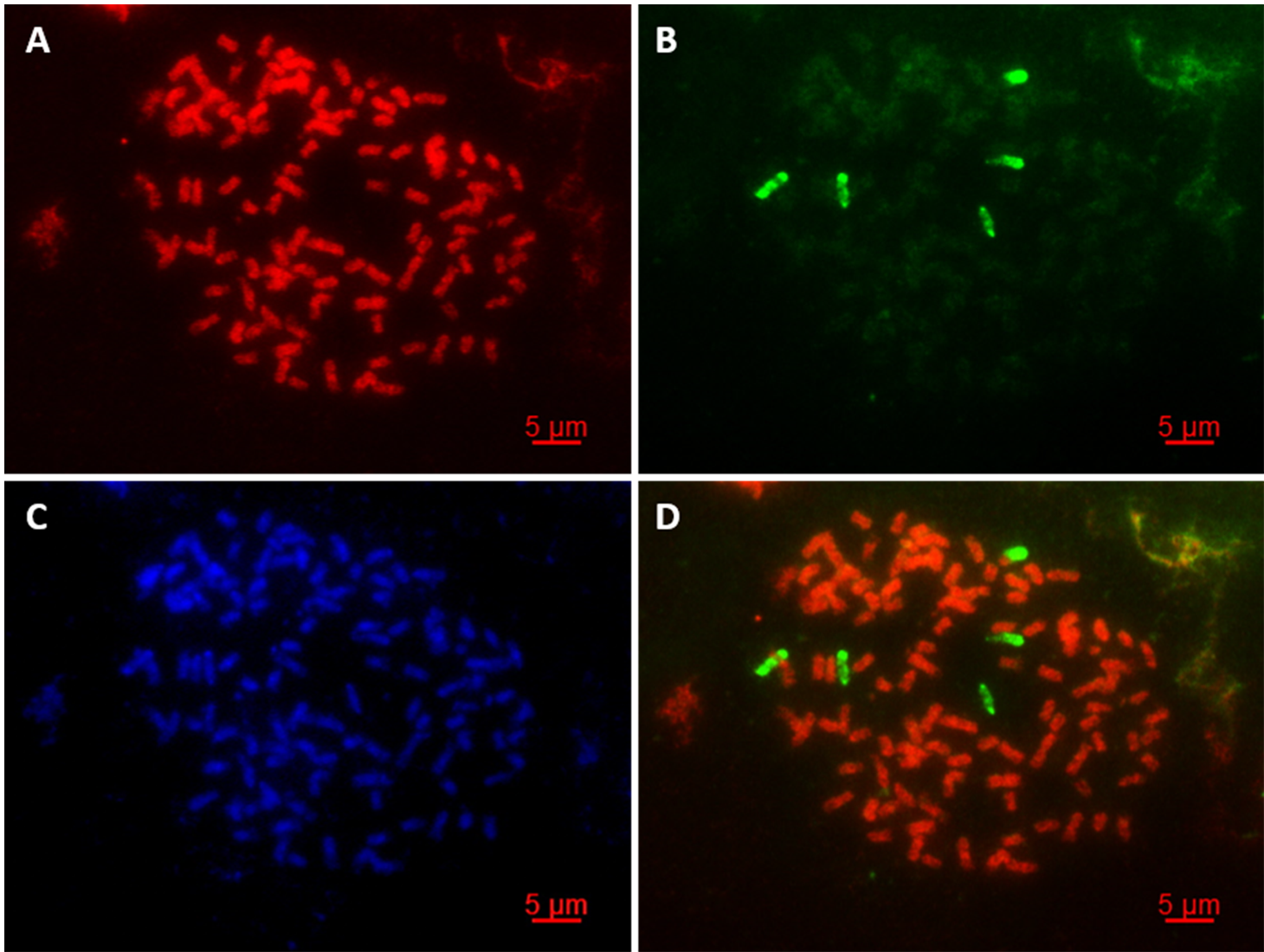


Fig. G. YCE06-140: 2n = 112 = 106S + 5E + S/E


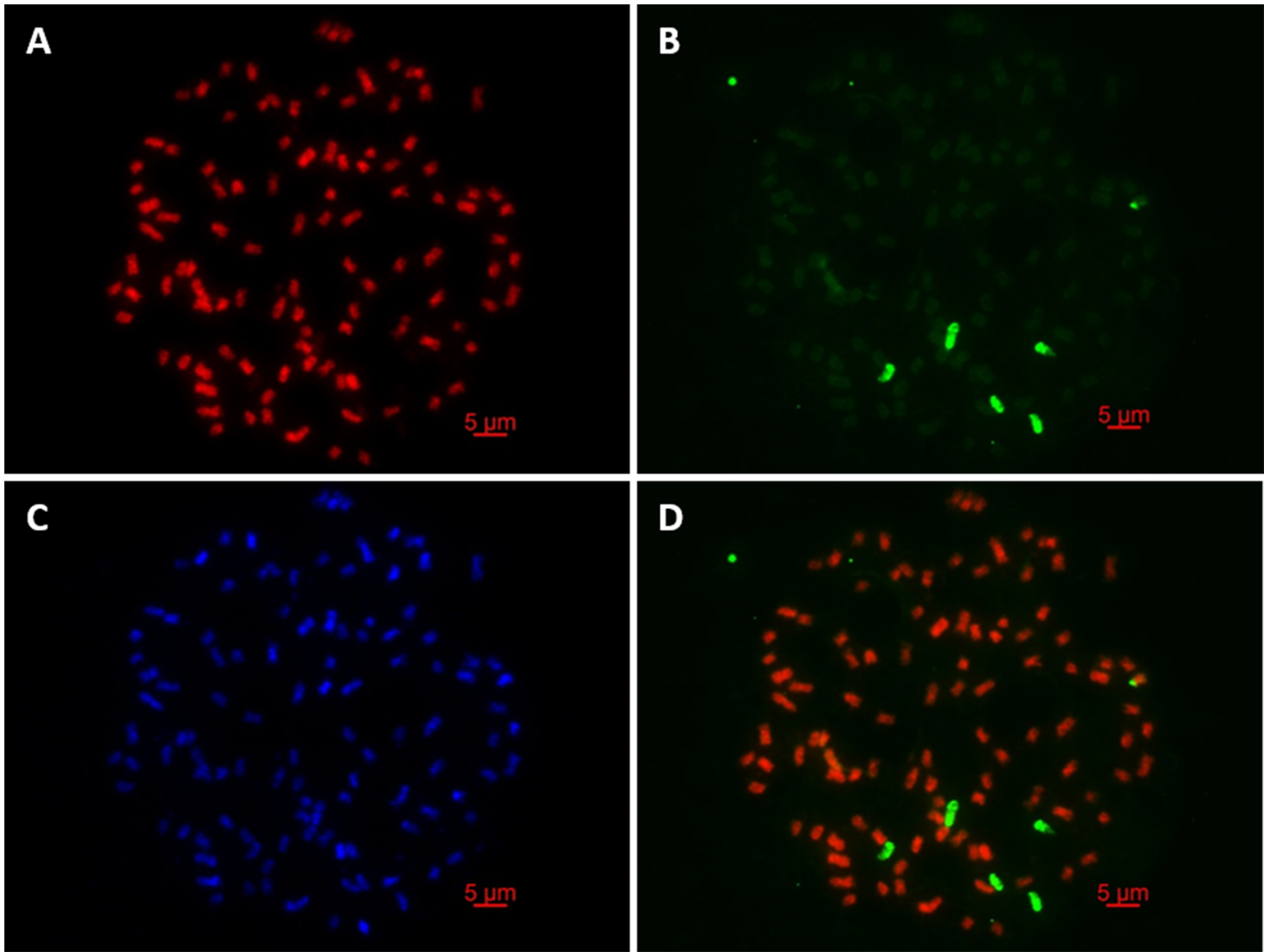


Fig. H. YCE06-166: 2n = 110 = 105S + 5E


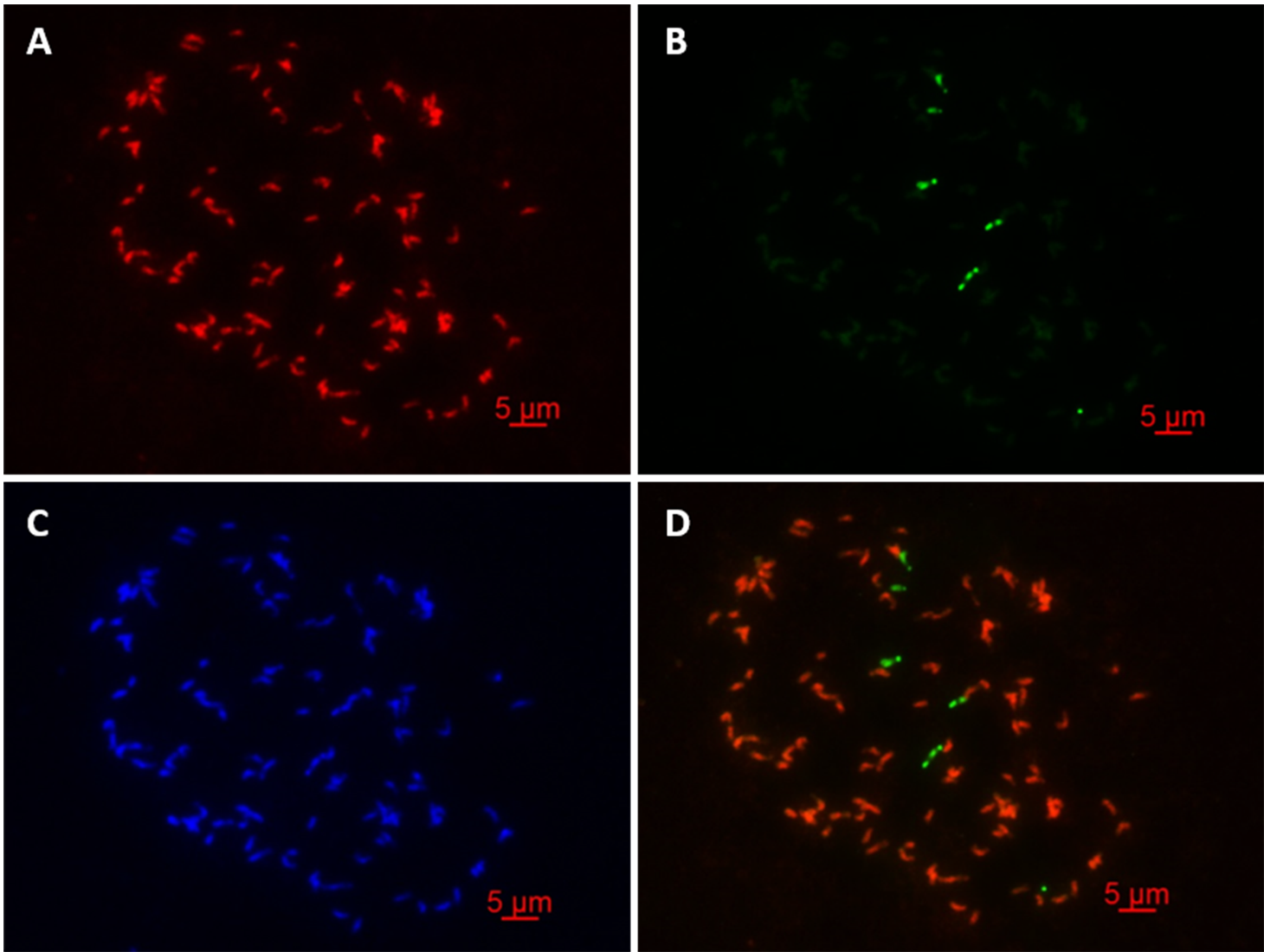

Supplement: S2 File — (A) Saccharum spp. chromosomes are visualized in red; (B) E. arundinaceus chromosomes are visualized in green; (C) All chromosomes are counterstained in blue; (D) A merged image is generated from the red and green channels. S and E indicate Saccharum spp. chromosome and E. arundinaceus chromosome, respectively. S/E and E/S indicate Saccharum spp. centromere with E. arundinaceus chromosome segment and E. arundinaceus centromere with Saccharum spp. chromosome segment, respectively. Scale bars: 5 μm. (DOC) [file pone.0133722.s002.doc]
